# *Vibrio cholerae* in the Environment: A Simple Method for Reliable Identification of the Species

**Published:** 2007-09

**Authors:** S. Baron, S. Chevalier, J. Lesne

**Affiliations:** Laboratoire d'Etude et de Recherche en Environnement et Santé, Ecole Nationale de la Santé Publique, Rennes, France

**Keywords:** Culture media, Diagnosis, Microbiology, Polymerase chain reaction, *Vibrio cholerae*, France

## Abstract

A simple screening and identification protocol was assessed for the efficient distinction of colonies of *Vibrio cholerae* species from others obtained on thiosulphate citrate bile salts sucrose agar after isolation from different environmental specimens. It was demonstrated here that the yellow colonies (sucrose-fermenting), which are able to grow on nutrient agar without added NaCl and which present a positive oxidase reaction, can be confidently considered as presumptive *V. cholerae*. Confirmation of the identification was carried out using the API 20E microtest and by species-specific *ompW*-based polymerase chain reaction: 809 of 925 isolates obtained by this screening procedure were identified as *V. cholerae* by API 20E and confirmed by PCR. The results showed that the direct use of the PCR-based method for the definite identification of the screened colonies gave better results than the API 20E method: of a selection of 100 isolates presumptively identified as *V. cholerae* according to the proposed screening procedure, all gave a positive result with PCR but only 94 were confirmed by API 20E. This protocol provides reliable identification of *V. cholerae* species and is adapted to the capabilities of routine clinical, food-testing and environmental microbiology laboratories.

## INTRODUCTION

More than 200 serogroups of *Vibrio cholerae* species have been identified, but only serogroup O1 and O139 are typically toxigenic and cause outbreaks of cholera, which is one of the most deadly enteric diseases acquired from contaminated water and food in many areas of Asia, Africa, and Latin America. Toxigenic *V. cholerae* are listed by the U.S. Centers for Disease Control and Prevention as category B bioterrorism agents (1) because they could be used for deliberately contaminating public water and food supplies. Unlike *V. cholerae* O1 and O139, non-O1/non-O139 serogroups typically do not produce cholera toxin, but some strains, the putative virulence genes of which have not yet been fully characterized, can cause the disease. The main clinical symptoms are gastroenteritis, skin infections, and also septicaemia (2). However, in most clinical laboratories, routine identification of *V. cholerae* is usually limited today to O1 and O139 serogroups, and serotyping is achieved directly after isolation of the bacterium on thiosulphate citrate bile salts sucrose (TCBS) agar selective media. But, in the case of septicaemia, isolation of *V. cholerae* from the bloodstream is not concerned with O1 and O139 serogroups. Furthermore, focusing on the agent of cholera while isolating *V. cholerae* from stool samples may lead to an underestimation of the part played by non-O1/non-O139 serogroups in the aetiology of gastroenteritis. Lastly, public-health studies, such as monitoring of water-quality and food-safety control, increasingly target the whole species as an emergent pathogen. All these warrant the need for rapid precise routine identification procedures in microbiology laboratories, for *V. cholerae* species as a whole.

This work addressed the demonstration of a simple, accurate, and quick method to identify colonies of *V. cholerae* obtained on TCBS agar from any isolation procedure (selective enrichment or direct plating). This method is composed of two steps: (a) a screening procedure using a few key taxonomically-important phenotypic traits, and (b) a species-specific PCR assay for definite identification.

## MATERIALS AND METHODS

### Collections of environmental isolates

The isolates used in this study were divided into three collections. All were collected from the Rance estuary (Brittany, France) and stored at −80°C in brain heart infusion with 10% glycerol added.

**Collection A:** Collection A comprised 93 isolates from three samples (two freshwater specimens and one aquatic sediment specimen) collected in August 2000. These specimens were incorporated (10% w/w) in saline alkaline peptone water (0.3% yeast extract, 1% peptone, 2% NaCl, and pH 8.6) (SAPW) and incubated at 41°C±1°C for 16−18 hours (3,4,5). Volumes of 0.1 mL of 10-fold dilutions of the enrichments were spread onto TCBS agar (Difco). All the yellow colonies present on TCBS agar were kept for the screening procedure, and all the presumptive isolates were biochemically identified with the API 20E system.

**Collection B:** Collection B was composed of 774 isolates from 115 samples (56 water samples, 46 sediment samples, and 13 cockle samples) collected during June-November 2000. These specimens were incorporated (10% w/w) in saline alkaline peptone water (0.3% yeast extract, 1% peptone, 2% NaCl, and pH 8.6) (SAPW) and incubated at 41°C±1°C for 16−18 hours (3,4,5). Only those isolates which were recognized by the screening procedure as presumptive *V. cholerae* were kept for definite identification.

**Collection C:** Collection C comprised isolates collected from a water sample in September 2003: 10 volumes of 0.1 mL of the water sample were directly (without any enrichment step) plated onto TCBS (DIFCO). One hundred presumptive isolates of *V. cholerae*, according to the screening procedure, were randomly selected and kept for identification by the three methods tested in this study.

### Identification

The identification methods tested in this study were the API 20E microtube test and the PCR, with two pairs of primers: one targeting the intergenic spacer region (ISR-based PCR) (6) and the other targeting the gene of the outer-membrane protein (*ompW*-based PCR) (7).

### Biochemical identification

For each isolate, one colony from an overnight culture on nutrient agar with 2% NaCl (NA_2_) was suspended in sterile saline water (0.85% NaCl) for inoculation of an API 20E strip (Biomerieux Industries, France) according to the instructions of the manufacturer. This was then incubated at 37°C for 24 hours. The series of 21 miniaturized biochemical tests was interpreted with the API 20E analytical profile index (version 5). The quality of the identification was based on two statistical values (% of identification [id] and t value). In this study, the identification results were classified as: (a) excellent identification when % of id was ≥99.9 and t value ≥0.75; (b) good identification when 90.0 ≥% of id <99.9%, and t was ≥0.25; and (c) low discrimination, when *V. cholerae* was not the only identification proposed.

### Molecular identification

**DNA extraction**: DNA extraction was done on overnight subculture on NA_2_ using the chloroform-phenol procedure (8). DNA extracts were treated with RNAse (1%) and dialyzed.

**PCR assays**: PCR amplification of the target DNA was carried out in a thermal cycler (Hybaid-PCR express) using 200-μL PCR tubes with a reaction mixture volume of 25 μL containing 3 μL of template DNA, 0.2 μL of each primer (100 μM), 2.5 μL of 2 mM dNTP, 0.125 μL (5 U/μL) of Taq polymerase (Eurobio), 2.5 μL of 10X reaction buffer, 1.25 μL of MgCl_2_ 50 M (Eurobio), and ultra-pure water. The sequences of the primers and the conditions of amplification used in this study are shown in Table 1.

**Table 1.** Characteristics of ISR-based PCR and ompW-based PCR used for identification of *Vibrio cholerae*

Name of primer	Target	Primer sequence (5′ → 3′)∗	Annealing†	Cycles‡	Length¶	Reference
ISR	16S–23S	AgT CAC TTA ACC ATA CAA CCC g	60–60	30	300	6
	rRNA	TTA AgC STT TTC RCT gAg AAT g				
OmpW	ompW	CAC CAA gAA ggT gAC TTT AAT TgT gs	64–30	30	588	7
		gAA CTT ATA ACC ACC gCg				

∗ The forward primer listed first, followed by the reverse primer

† Annealing conditions: temperature in °C—time in seconds

‡ Cycles=Number of repetitions for amplification of the dissociation, annealing, and elongation steps

¶ Length of amplification product in base pairs

ISR=Intergenic spacer region; PCR=Polymerase chain reaction

PCR products were separated by agarose gel electrophoresis, followed by ethidium bromide staining. For each PCR reaction, a reagent blank was run, where the DNA template was replaced by ultra-pure water. DNA of a strain of *V. cholerae* O1, Classical, Inaba (Institut Pasteur—CNRVC 940147) was run as a positive control. When an isolate gave a negative result, the PCR reaction was repeated once.

As the authors had already tested specificity, only the absence of cross-reaction of the two sets of primers with *V. mimicus* was checked on 14 environmental strains of *V. mimicus* (provided by the LERES collection). The quality of DNA extraction for each strain was verified by amplification of 16S rDNA with the 27F and 1492R primers (9).

### Screening procedure

The screening procedure was based on phenotypic traits. The sucrose-fermenting colonies on TCBS were transferred onto nutrient agar without the addition of NaCl (NA_0_) for growth testing and were then submitted to an oxidase test (Bactident strip). All sucrose-fermenting isolates, able to grow on NA_0_ and oxidase-positive, were considered to be presumptive isolates of *V. cholerae* and were then tested for culture purity on NA_2_.

### Experimental plan

The three collections were used for testing the reliability of the screening procedure. Collection C was also used for comparing the techniques of the definite identification of presumptive isolates. Therefore, the same overnight culture on NA_2_ of each strain was used in parallel for biochemical and molecular identification.

## RESULTS

### Reliability of the screening procedure

In collection A, 51 of the 93 sucrose-fermenting colonies on TCBS agar were able to grow on NA_0_ and gave an oxidase-positive reaction (Table 2). Ninety-six percent of these presumptive isolates gave excellent identification as *V. cholerae* with the API 20E system and 4% good identification. The remaining 42 strains were not identified.

**Table 2:** Reliability of the screening procedure tested with the API 20E system (environmental isolates of collection A)

Screening phase					Identification phase	
Criterion	Sucrose-positive	Growth on NAo	Oxidase-positive	API 20E profile	Quality of identification (% of id∗/t index†)	
Sample	No. of colonies (%)					No. of colonies (%)	
Running freshwater	21	13	11 (52.4)	5347124	Excellent (99.9/1.00)	10 (90.9)
				5147124	Excellent (99.9/0.92)	1
Lentic freshwater	32	32	32 (100.0)	5347124	Excellent (99.9/1.00)	31 (96.9)
				5346124	Good (99.6/0.96)	1
Sediment	40	8	8 (20.0)	5347124	Excellent (99.9/1.00)	7 (87.5)
				5347125	Good (99.4/0.79)	1
Total	93	53	51 (54.8)	5347124	Excellent (99.9/1.00)	48 (96.0)

∗ % of id: Percentage of identification—an estimate of how closely the profile corresponds to the taxon relative to all the other taxa in the database

† t index: An estimate of how closely the profile corresponds to the most typical set of reactions for each taxon; its value varies between 0 and 1 and is inversely proportional to the number of atypical sets

Table 3 shows the results of the identification of the 774 presumptive isolates of collection B with the API 20E system: 662 isolates were correctly identified as *V. cholerae*, and 593 of these isolates were excellently identified. Of the 112 remaining isolates, eight were identified as *Aeromonas*, and 49 could not be identified by the API 20E system.

**Table 3:** Realibiliy of the screening procedure assessed with the API 20E system (774 environmental isolates of collection B)

		Excellent identification	593
	*Vibrio cholerae* (692)	Good identification	69
		Low discrimination	30
		*V. alginolyticus*	5
		*V. vulnificus*	1
Presumptive isolates (774)		*V. mimicus/V. vulnificus*	2
	Other species (33)	*Aeromonas*	8
		*Shewanella putrefaciens*	12
		*Chryseobacterium meningosepticum*	1
		*Pseudomonas shigelloides*	4
	Unknown		49

Lastly, in the third set of data (collection C), 96% of the presumptive isolates were identified as *V. cholerae* in the API 20E system, with 84% having an excellent identification (Table 4).

**Table 4:** Comparison of definitive identification of 100 presumptive isolates (collection C) by 3 methods in parallel

No. of strains	Molecular identification		Biochemical identification	
	ISR-based PCR	ompW-based PCR	API 20E profile	Quality of identification (% of id∗;/t index†)
78	+	+	5347124	Excellent (99.9/1.00)
6	-	+	5347124	Excellent (99.9/1.00)
4	+	+	5346124	Good (99.6/0.98)
8	+	+	5247124	Good (90.1/0.75)
4	+	+	7347124	Low discrimination (82.9/0.67)

∗ % of id: see Table 1;

† t index: see Table 1

The enrichment step did not seem to be the key factor since the confirmation rate of the presumptive identifications by the API 20E system was approximately the same for collection B and C isolates.

The proposed screening procedure seemed to be very efficient since the identification confirmation rate was more than 85%, whatever the nature of the specimen or its treatment before spreading on TCBS.

### Comparison of the identification methods

The confirmation rate by ISR-based PCR of good and excellent identifications with API 20E was 99.2% (of 762 tested in collections B and C, 756 had confirmed identifications).

Table 4 shows the results obtained from collection C isolates, presumptively identified as *V. cholerae*. The identification was confirmed by the three methods for 90 of 100 isolates. For the 10 remaining isolates, the identification was confirmed at least by two methods: four isolates gave a positive reaction with the two PCR assays, whereas identification by API 20E gave low discrimination; for the six other isolates, the identification by API 20E was excellent but only the *OmpW*-based PCR gave a positive reaction.

Thus, these results showed that PCR can be used for checking a doubtful biochemical identification (6 isolates) and that the *OmpW*-based PCR seemed to be the best of the two PCR assays.

Indeed, compared to API identification, ISR-based PCR, used as a reference method, gave 6% false positives, whereas *OmpW*-based PCR gave none. Second, 94% of the presumptive identifications were confirmed by the two PCR reactions compared to 100% by the *OmpW*-based PCR only.

The specificity of these PCR-based confirmed identifications was tested for closely-related *V. mimicus* species. Vieira *et al.* showed that the *V. cholerae* amplicon obtained by ISR-based PCR was present in 11% (3/26) of *V. mimicus* isolates (10), and Nandi *et al.* used only six strains of *V. mimicus* to prove the specificity of *V. cholerae* identification using a *OmpW*-based PCR (7). In this study, 14 strains of *V. mimicus* were tested and gave a negative result with both ISR-based PCR and *OmpW*-based PCR.

## DISCUSSION

### Performance of screening of isolates after isolation on TCBS agar

Routine screening of isolates on any selective agar relies on the capacity of selection of the target bacteria and on the choice of a few relevant taxonomic traits for presumptive identification. TCBS agar is commonly recommended for the isolation of *Vibrio* spp. (11,12). It eliminates non-bile salt tolerant species and remains the best agar for its ability to isolate vibrios from their natural estuarine environment (13).

Our results are consistent with the proposition of Muic *et al.* (14), saying that the use of TCBS agar as a selective medium is probably a key precondition for making any proposed selection of traits effective for good presumptive identification of vibrios.

In this study, the biochemical traits were chosen among 13 readily-determinable ones proposed by Baumann *et al.* (15). Three ordered traits (sucrose fermentation, non-requirement of added Na^+^ for growth, and presence of oxidase) are sufficient to distinguish *V. cholerae* from the other species of *Vibrio*. Sucrose non-fermenting species are not taken into consideration, and, thus, the related *V. mimicus* species is eliminated at the beginning. Growth on nutrient agar without added NaCl eliminated most sucrose-positive halotolerant or halophilic vibrios that may be important in differential diagnoses (*V. alginolyticus, V. metschnikovii*, and *V. fluvialis* and to a lesser extent *V. furnissii, V. cincinnatiensis, V. anguillarum*, and *V. carchariae*). Moreover, a oxidase-positive reaction eliminated *V. metschnikovii*.

While these three taxonomic traits are insufficient to definitively identify the isolates as *V. cholerae*, they do represent minimal traits which all members of the species must have. Thus, any isolates which do not meet these minimal criteria are deemed not to be *V. cholerae*.

Our results, which are based on a very large number of environmental isolates (collection A and collection B) emphasize the fact that the efficiency of the proposed screening procedure is reliable for environmental monitoring. Even if one notices that some rare variants (sucrose-negative or inability to grow whithout added NaCl) of *V. cholerae* may be lost by this screening scheme, the option remains relevant for routine screening.

Choopun *et al.* proposed another selection of traits for the rapid presumptive identification of V. chole-rae from aquatic environments (arginine dihydrolase activity-negative and esculin hydrolysis-negative—two expensive and relatively difficult tests) (16). Although esculin hydrolysis has been proposed by Baumann *et al.* as a key taxonomic trait (15), it is not easy to interpret the test without a fluorometer, which is absent in most laboratories.

### Choice of a confirmative identification method

The API 20E system is indeed considered an acceptable method for the identification of the more commonly-occurring members of the family *Vibrionaceae* (17,18), even if there are very few reports expressly concerned with the ability of commercial systems to identify members of the genus *Vibrio* (19).

Besides conventional biochemical identification, alternative molecular methods, such as PCR or hybridation of colonies by labelled probes, are available for the confirmation of presumptive identifications.

Colony-blot probing on selective agar, such as alkaline nutrient agar without added NaCl, has been proposed for the isolation of *V. cholerae* (20). This alternative process does not need presumptive biochemical identification, but cannot use TCBS agar selectivity. Moreover, its application is limited to the case of brackish water monitoring. Another common alternative method is PCR, which is now used by reference laboratories as the most reliable means of routine identification.

Only two of four pairs of primers which were available for the identification of *V. cholerae* species were compared. The one developed by Chun *et al.* which targets a highly-conserved intergenic spacer region of the 16S−23S rRNA gene sequences (6), and one of the two developed by Nandi *et al.* which targets the *OmpW* gene (7). The primers targeting the haemolysin genes of *V. cholerae* were not included in our comparison (21) because, in our collection of environmental strains, haemolysin genes were often absent (unpublished data). The numerous other primers, described in the literature, target cholera virulence-associated genes and do not allow the identification of *V. cholerae* species. They are used for distinguishing between toxigenic and non-toxigenic *V. cholerae*. Moreover, in most *V. cholerae* non-O1/non-O139 strains, cholera virulence-associated genes are absent.

In our work, the use of three identification methods in parallel on a large number of isolates (collection C) was able to illustrate the advantages of PCR for the confirmation of presumptive identifications. Based on our data (very few API−, PCR+ results and no API+, PCR− results), we can say that there is a little difference between the tested biochemical and the molecular methods used for confirming the presumptive identifications born of the screening procedure. Previous observation on this had never been carried out using such a large number of environmental isolates. However, identification by PCR should be the preferred method as it is known to be quicker to perform and more accurate. Leroux *et al.* obtained different results in a similar study, with no API−, PCR+ and a significant proportion of API+, PCR− results, but they used another pair of primers targeting the *OmpW* gene (22).

In this study, PCR reactions targeting ISR and the *OmpW* gene were compared on a large collection of environmental isolates of *V. cholerae* and a significant number of *V. mimicus* isolates, which had never been done before. Based on our results, it can be concluded that *OmpW*-based PCR should be preferred to ISR-based PCR and that there was no evidence of cross-reaction with the closely-related *V. mimicus* species.

In conclusion, the proposed simple procedure for the identification of *V. cholerae* after isolation on TCBS agar is based upon a combination of phenotypic and genotypic testing methods, which is recommended for the identification of any taxon (23). This is suited to microbiological monitoring of the aquatic environment which requires techniques with higher resolution.

The screening of presumptive isolates on TCBS agar is quick, cheap, and easy to perform, and the reading of results are clear-cut and reliable. Also, the associated identification method by *OmpW*-based PCR is quicker and slightly more sensitive than the API 20E system.

As a whole, the proposed procedure is also reasonable with respect to time consumption and expense. It is also adapted to the capabilities of a routine microbiology laboratory (clinical, environmental and food-testing) and is easily adaptable to the workflow in such laboratories. All these characteristics are beneficial to public health.
